# Correction: Prevalence of chronic kidney disease among young people living with HIV in Sub Saharan Africa: A systematic review and meta-analysis

**DOI:** 10.1371/journal.pone.0326624

**Published:** 2025-06-17

**Authors:** Esther M. Nasuuna, Nicholus Nanyeenya, Davis Kibirige, Jonathan Izudi, Chido Dziva Chikwari, Robert Kalyesubula, Barbara Castelnuovo, Laurie A. Tomlinson, Helen A. Weiss

Fig 2 is uploaded incorrectly. Please see the correct Fig 2 here.

**Fig 2:**
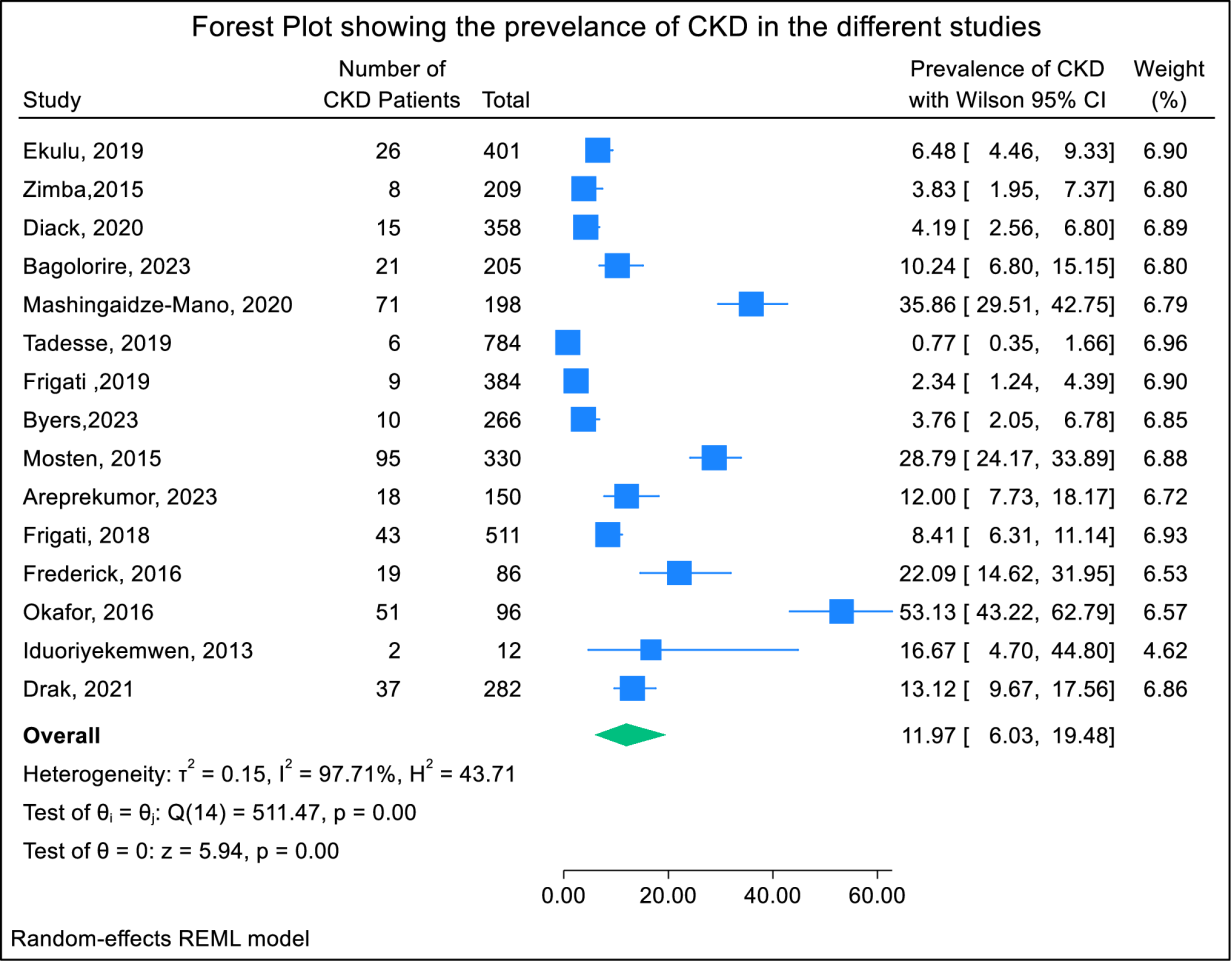
Forest plot of studies reporting the prevalence of CKD among YPLHIV in SSA.
