# Supplementary material for: Prevalence of chronic kidney disease among young people living with HIV in Sub Saharan Africa: A systematic review and meta-analysis
Source: PLoS One. 2024 Nov 4;19(11):e0301954. doi: 10.1371/journal.pone.0301954 (PMC11534254; doi:10.1371/journal.pone.0301954)
Supplement: S4 Appendix — (DOCX) [file pone.0301954.s004.docx]

| **No** | **First author, year** | **Country** | **Region of Africa** | **Population** | **Study design** | **Age** | **Control group** | **Sex % of females** | **Sample size** | **No of CKD patients** | **Prevalence (95% CI)** | **Disease definition or diagnosis** | **Equation used** | **Biomarker used** | **Follow up** |
| --- | --- | --- | --- | --- | --- | --- | --- | --- | --- | --- | --- | --- | --- | --- | --- |
| 1 | Diack 2020 | Burkina Faso, Côte d’Ivoire, Burundi, Senegal, Mali and Cameroon | Western and Eastern | 0 to 18 yrs treated with TDF containing regimens for at least 1.5 years | Cross sectional | Median 15.5 IQR (14 to 16.8 )years | No | 51% | 358 | 15 | 4.1% (2-7%) | eGFR 30- 60ml/min/1.73m^2^ | 2009 Schwartz | Serum Creatinine measured by enzymatic method | Single |
| 2 | Ekulu 2019 | Democratic Republic of Congo | Central | treated with non-renal toxic drugs followed in the HIV clinics. | Cross sectional | Mean age in years 11.6 +/-4.1 | Yes (HIV-from the general population) | 51.1% | 401 | 26 | 6.5% (4-9%) | eGFR <60ml/min/1.73m^2^ | Schwartz | Serum Creatinine measured by enzymatic method | Single |
| 3 | Tadesse 2019 | Ethiopia | Eastern | Perinatally HIV infected on ART> 6 months | Cohort | Median 12 years (IQR 8-14) | No | 46.6% | 784 | 6 | 0.8% (0.2-1%) | eGFR 60-90 ml/min/1.73m^2^ | MDRD | Serum Creatinine | 6 monthly follow up |
| 4 | Frigati 2019 | South Africa | Southern | Perinatally infected on ART for more than 6 months | Cross sectional | 9 to 14 years with mean 12 years SD 1.7 | Yes (HIV-matched on age, ancestry) | 47.7% | 384 | 9 | 2.3% (1.1-4.4%) | eGFR<90 ml/min/1.73m^2^ | Modified Schwartz | Serum Creatinine measured by enzymatic method | Single |
| 5 | Mashingaidze-Mano, 2020 | Zimbabwe | Southern | ON TDF regimen for >6 months | Cross sectional | Median 15 (IQR 13-16) | No | 44.9% | 198 | 71 | 35.9% (29-43%) | eGFR<90 ml/min/1.73m^2^ | Schwartz | Serum and urine creatinine measured using the modified Jaffe method. | Single |
| 6 | Frederick 2016 | Tanzania | Eastern | Both ART naïve and experienced | Cross sectional | 10 to 14 years | No | 44.6% | 86 | 19 | 22.1% (13.9-32.3%) | ACR >30mg/g | Not applicable | Creatinine | Single |
| 7 | Frigati 2018 | South Africa | Southern | On ART for more than 6 months | Cross sectional | 9 to 14 years | Yes (HIV negative) | 48.7% | 511 | 43 | 8.4% (6.1-11.2%) | ACR >30mg/g | Not applicable | Serum creatinne measured by enzymatic method | Single |
| 8 | Iduoriyekemwen, 2013 | Nigeria | Western | ART experienced YPLHIV attending ART clinic | Cross sectional | 12 to 17 years | No | 40% | 12 | 2 | 16.7% (2-48%) | Proteinuria 1+ OR microalbuminuria of ≥20 mg OR eGFR <60ml/min/1.73m^2^ | Schwartz | Serum creatinine | Single |
| 9 | Okafor 2016 | South South Nigeria | Western | ART naïve presenting to ART clinic for first time | Cross sectional | 18 to 29 years | No | 58% | 96 | 51 | 53.1% (43-63%) | eGFR <60 ml/min/1.73m^2^ | Modification of Diet in Renal disease (MDRD) | Creatinine | Single |
| 10 | Drak, 2021 | Zimbabwe | Southern | ART naïve presenting to ART clinic for first time | Cross sectional | Median 14.3 years (14.1 -14.5) | No | 55% | 282 | 37 | 13.1% (9-17.6%) | GFR<90ml/min/1.73m^2^ | Full age spectrum formula | Creatinine | Single |
|  |  |  |  |  |  |  |  |  | 209 | 15 | 7.2% (4-11.4%) | Proteinuria of 1+ on dipstick |  |  | Single |
| 11 | Zimba 2015 | Zambia | Southern | HIV infected 18 months to 16 years | Cross sectional | mean 9.3 years (3.84) | No | 50.2% | 209 | 8 | 3.8% (1.7-7.4%) | eGFR<60ml/min/1.73m^2^ | Schwartz | Creatinine | Not mentioned |
|  |  |  |  |  |  |  |  |  | 209 | 17 | 8.1% (4.8-12.7%) | Proteinuria of 1+ on dipstick | Not applicable | Not applicable | Not mentioned |
| 12 | Mosten 2015 | Tanzania | Eastern | HIV infected 0 to 17 years. | Cross sectional | Mean 10 years (4-18) | No | 44.8% | 330 | 95 | 28.8% (23.9-34%) | Micro | Not applicable | Not applicable | One month apart |
|  |  |  |  |  |  |  |  |  |  |  |  | albuminuria 20 -200 mg/L |  |  |  |
| 13 | Bagoloire 2023 | Uganda | Eastern | HIV infected on ART | Cross sectional | 10-17 years | No | 50.6% | 205 | 21 | 10.2 (6.8-15.2) | eGFR<60ml/min/1.73m^2^ | Not mentioned | Creatinine | Single |
| 14 | Areprekumor 2023 | Nigeria | Western | 15 to 18 years on ART | Cross sectional | 15 to 18 years | Yes | 50.0% | 150 | 18 | 28.1 (17.5-40.8) | Microalbuminuria 20-200mg/l | Not applicable | Not applicable | Single |
| 15 | Byers, 2023 | Zimbabwe | Southern | ART naïve presenting to ART | Cohort | 12-17 years | No | 55.0% | 266 | 10 | 3.8 (1.8-6.8) | GFR<90ml/min/1.73m^2^ | Full age spectrum | Creatinine | 4 years |
