# Supplementary material for: Prevalence of chronic kidney disease among young people living with HIV in Sub Saharan Africa: A systematic review and meta-analysis
Source: PLoS One. 2024 Nov 4;19(11):e0301954. doi: 10.1371/journal.pone.0301954 (PMC11534254; doi:10.1371/journal.pone.0301954)
Supplement: Attachment — Submitted filename: Response to reviewers summary.docx [file pone.0301954.s006.docx]

Response to Reviewer’s comments

Reviewers' comments:

**Comments to the Author**

1. Does this manuscript meet PLOS Global Public Health’s [publication criteria](https://journals.plos.org/globalpublichealth/s/criteria-for-publication)? Is the manuscript technically sound, and do the data support the conclusions? The manuscript must describe methodologically and ethically rigorous research with conclusions that are appropriately drawn based on the data presented.

Reviewer #1: Partly

2. Has the statistical analysis been performed appropriately and rigorously?

Reviewer #1: Yes

3. Have the authors made all data underlying the findings in their manuscript fully available (please refer to the Data Availability Statement at the start of the manuscript PDF file)?

The [PLOS Data policy](https://journals.plos.org/globalpublichealth/s/data-availability) requires authors to make all data underlying the findings described in their manuscript fully available without restriction, with rare exception. The data should be provided as part of the manuscript or its supporting information or deposited to a public repository. For example, in addition to summary statistics, the data points behind means, medians and variance measures should be available. If there are restrictions on publicly sharing data—e.g. participant privacy or use of data from a third party—those must be specified.

Reviewer #1: Yes

4. Is the manuscript presented in an intelligible fashion and written in standard English?

PLOS Global Public Health does not copyedit accepted manuscripts, so the language in submitted articles must be clear, correct, and unambiguous. Any typographical or grammatical errors should be corrected at revision, so please note any specific errors here.

Reviewer #1: Yes

5. Review Comments to the Author

Please use the space provided to explain your answers to the questions above. You may also include additional comments for the author, including concerns about dual publication, research ethics, or publication ethics. (Please upload your review as an attachment if it exceeds 20,000 characters)

Reviewer #1: This study by Nasuuna et al reports prevalence of CKD in young people (age 10 to 24 years) living with HIV in sub-Saharan Africa (SSA). A meta-analysis could not be performed due to high heterogeneity across identified studies. This study included 13 studies identified across SSA.

I understand some of the challenges the authors have had to contend with - especially that of low quantity and quality of studies from the SSA region on this subject. However, I think there are several major issues that need to be addressed in this study:

1. The definition of CKD is usually taken as eGFR <60ml/min, confirmed on a repeat test. The studies included in this study, had varying ways of assessing kidney function and reporting of kidney dysfunction. Although the authors report heterogeneity for not pooling the data, this makes the findings of the study very difficult to uphold. especially the observed wide range of CKD prevalence (0.8% to 53.1%). This, I believe is a major problem with this study. Perhaps the study could be limited to those with a uniform definition of CKD or maybe just those that repeated the test after a period of time.

**Response:** There were very few studies that report on kidney function among young people living with HIV (YPLHIV) in SSA. Because of the lack of standardisation, they all used different criteria to diagnose CKD different from the American recommended definition of eGFR<60ml/min on two separate occasions. To overcome this and despite the high heterogeneity we did a meta-analysis to help us understand the data further. We did subgroup analysis, by CKD definition, by whether or not the measurements were repeated, by region and, by sample size. See the improved paper.

2. In one study (Tadesse et al), eGFR was reported to have declined by 35.6 ml/min. However, the chart (Figure 2) shows reduced GFR from over 400 ml/min at time zero to over 200 ml/min after 18 months. How did the authors account for these in defining CKD or including this study?

**Response**: In the Tadesse study, which was a cohort study that run for 18 months, we considered the measurements done at the 6 month follow up as this satisfied the criteria for CKD diagnosis. Since the paper was focused on prevalence of CKD, this was sufficient. We did not report on the fall in GFR over time.

3. How were the authors able to be sure abnormal kidney function reported in some studies were not cases of acute kidney injury?

**Response**: We cannot be sure that some studies did not report acute kidney injury. However, the prevalence is still relevant as chronic kidney disease starts out as acute kidney injury and those who had acute kidney injury are more prone to chronic kidney disease. We did report the prevalence for the studies that repeated the tests so that can also be considered when interpreting the findings of the systematic review and meta-analysis.

4. Although the age of young people to be included is mentioned as 10-24 years, there are studies that included people younger that 10 years (e.g., Tadesse et al [2019], Frigati et al [2018 and 2019], Zimba et al [2015]) and those older than 24 years (Okafor et al 2016). How do the authors account for this?

**Response:** Most of the studies did not show prevalence by our specific age band and we therefore included studies whose participants had a mean or median age within our age band as well as those who had participants within that age band. See the eligibility criteria in the methods section. In the limitations, we mentioned the lack of age disaggregated data as a limitation. However, we believe that this review still gives a relatively good estimate of the prevalence of CKD within a very understudied population that further studies can benchmark off of.

6. PLOS authors have the option to publish the peer review history of their article ([what does this mean?](https://journals.plos.org/globalpublichealth/s/editorial-and-peer-review-process#loc-peer-review-history)). If published, this will include your full peer review and any attached files.


**Do you want your identity to be public for this peer review?** If you choose “no”, your identity will remain anonymous but your review may still be made public.


For information about this choice, including consent withdrawal, please see our [Privacy Policy](https://www.plos.org/privacy-policy).

Reviewer #1: No
