# Supplementary material for: Prevalence of chronic kidney disease among young people living with HIV in Sub Saharan Africa: A systematic review and meta-analysis
Source: PLoS One. 2024 Nov 4;19(11):e0301954. doi: 10.1371/journal.pone.0301954 (PMC11534254; doi:10.1371/journal.pone.0301954)
Supplement: Attachment — Submitted filename: Response to reviewers July 2024.docx [file pone.0301954.s008.docx]

Response to reviewers

Please do the following and re-submit the manuscript.
1. Please remove the urinary NGAL study from the list of articles included, as suggested by one of the reviewers. Using an NGAL study simultaneously with creatinine-based GFR does not appear appropriate. Repeat the analysis without the NGAL study. Of course, the NGAL study can be referenced in the discussion section while highlighting the need for early CKD diagnosis.

Response: the NGAL paper has been removed from the analysis. The systematic review now includes 15 and not 16 papers. This has been reflected in all tables, figures and in the text. See attached manuscript.

2. Kindly convert Appendix 4 from EXCEL to MS Word format and resubmit

Response: appendix 4 has been converted to word and is attached as a word document.
